# The functional readthrough extension of malate dehydrogenase reveals a modification of the genetic code

**DOI:** 10.1098/rsob.160246

**Published:** 2016-11-23

**Authors:** Julia Hofhuis, Fabian Schueren, Christopher Nötzel, Thomas Lingner, Jutta Gärtner, Olaf Jahn, Sven Thoms

**Affiliations:** 1 Department of Pediatrics and Adolescent Medicine, University Medical Center Göttingen, University of Göttingen, 37075 Göttingen, Germany; 2 Microarray and Deep Sequencing Core Facility, University Medical Center Göttingen, University of Göttingen, 37077 Göttingen, Germany; 3 Proteomics Group, Max Planck Institute of Experimental Medicine, 37075 Göttingen, Germany

**Keywords:** translational readthrough, genetic code, peroxisome, MDH1x, LDHBx, redox shuttle

## Abstract

Translational readthrough gives rise to C-terminally extended proteins, thereby providing the cell with new protein isoforms. These may have different properties from the parental proteins if the extensions contain functional domains. While for most genes amino acid incorporation at the stop codon is far lower than 0.1%, about 4% of malate dehydrogenase (MDH1) is physiologically extended by translational readthrough and the actual ratio of MDH1x (extended protein) to ‘normal' MDH1 is dependent on the cell type. In human cells, arginine and tryptophan are co-encoded by the MDH1x UGA stop codon. Readthrough is controlled by the 7-nucleotide high-readthrough stop codon context without contribution of the subsequent 50 nucleotides encoding the extension. All vertebrate MDH1x is directed to peroxisomes via a hidden peroxisomal targeting signal (PTS) in the readthrough extension, which is more highly conserved than the extension of lactate dehydrogenase B. The hidden PTS of non-mammalian MDH1x evolved to be more efficient than the PTS of mammalian MDH1x. These results provide insight into the genetic and functional co-evolution of these dually localized dehydrogenases.

## 1. Introduction

Decoding of stop codons as sense codons is known as translational readthrough or stop codon suppression and was first described in viruses [1–3]. Later, prokaryotic, eukaryotic and additional viral genes were discovered that are naturally readthrough [4–7]. Bioinformatic approaches and ribosome profiling in *Drosophila* identified genes displaying translational readthrough [8–10], and readthrough in mammals was also reported for several singular genes [5,6,8,9,11–17].

How readthrough is controlled at the ribosome is not clear, but the first *trans*-acting factors interacting with the termination complex are being uncovered [18,19]. On the level of the RNA, readthrough efficiency is determined by the stop codon itself and by elements more distantly located on the mRNA [12,20–24]. Moreover, the nucleotides surrounding the stop codon, referred to here as the stop codon context (SCC), have an impact on readthrough efficiency [2,25–29]. Recently, we developed a linear regression model for a genome-wide *in silico* readthrough analysis focusing on translational readthrough that depends on the SCC [15]. This model computes a readthrough propensity (RTP) score for the SCC of every human transcript. Using the regression coefficients of this model, we derived a consensus for high-translational readthrough in mammals: UGA CUA (G) (stop codon underlined) [15]. This high-readthrough SCC raises the readthrough rate by at least one order of magnitude, from less than 0.1% to more than 1% [14–16].

It is noteworthy that SCC-dependent readthrough can be obtained without pharmacological modification of the ribosomal stop fidelity. Drug-induced increase in readthrough, on the other hand, is considered a therapeutic option in some genetic diseases that are caused by a premature stop codon mutation [30,31]. Most of the drugs that are currently being tested are aminoglycosides or their derivatives. These bind to the small subunit of the ribosome and reduce discrimination of near-cognate tRNAs [32].

Peroxisomes are cellular organelles involved in fatty acid β-oxidation and degradation of hydrogen peroxide [33]. In mammalian cells, peroxisomes degrade complex fatty acids such as branched chain fatty acids and very long-chain fatty acids, and also synthesize bile acids [34]. Most luminal proteins contain a peroxisomal targeting signal type 1 (PTS1) at the very C-terminus, for example the canonical tripeptide serine-lysine-leucine (SKL) [35]. However, even the classical SKL tripeptide is not always a PTS1 as more than the three terminal amino acids are involved in the targeting process [36]. The substrates themselves may or may not be oligomers during import [37,38].

*Functional translational readthrough* gives rise to new protein isoforms with biological functions distinct from that of the original protein. Ribosomal readthrough in fungi, for example, leads to peroxisomal targeting of some glycolytic enzymes due to the presence of hidden targeting signals in the readthrough extensions [39]. In our previous work, we identified physiologically relevant genes regulated by translational readthrough by combining the RTP scores with a search for PTS in the C-terminal extensions. LDHB, the heart subunit of lactate dehydrogenase (LDH), was found to have the highest combined score, i.e. the highest combined score of RTP value and probability of containing a PTS in the extension [15]. Readthrough of the LDHB SCC and the full-length gene was confirmed experimentally and it was shown that the readthrough-extended isoform of LDHB is imported into peroxisomes via the hidden PTS1 [15,16].

RTP prediction yielded 57 human readthrough candidates with the high-readthrough consensus [15,17]. Malate dehydrogenase 1 (MDH1) showed the highest RTP. MDH1 was also detected as a potential readthrough protein by phylogenetic analysis [14] and ribosome profiling in human foreskin fibroblasts [8]. MDH1 mediates reversible conversion of malate and NAD^+^ to oxaloacetate and NADH and at least two isoforms are present in eukaryotic cells. Mitochondrial MDH1 is involved in the citric acid cycle, and the cytoplasmic form supports the malate–aspartate shuttle across the mitochondrial inner membrane [40]. MDH1, like LDHB, is extended by translational readthrough and transported into peroxisomes via a hidden PTS1 [16]. Interestingly, earlier proteomic analysis had already found MDH1 in mammalian peroxisomes [41,42].

In this study, we analyse the stop codon readthrough of MDH1. We show that readthrough is dependent on the SCC, but not on the subsequent 50 nucleotides. By employing a quantitative assay, we show that MDH1 readthrough is tissue-specific and exceeds LDHB readthrough in all tested cell types. We demonstrate that the MDH1x (extended protein) stop codon encodes tryptophan and arginine. This natural stop codon recoding, which is stimulated by the SCC, constitutes a modification of the genetic code in humans. Furthermore, we provide evidence that the higher degree of conservation of MDH1 readthrough in comparison to LDHB readthrough co-evolved with the targeting signal strength of their respective PTS1.

## 2. Results

### 2.1. Analysis of the malate dehydrogenase stop codon readthrough

**Figure 1.**
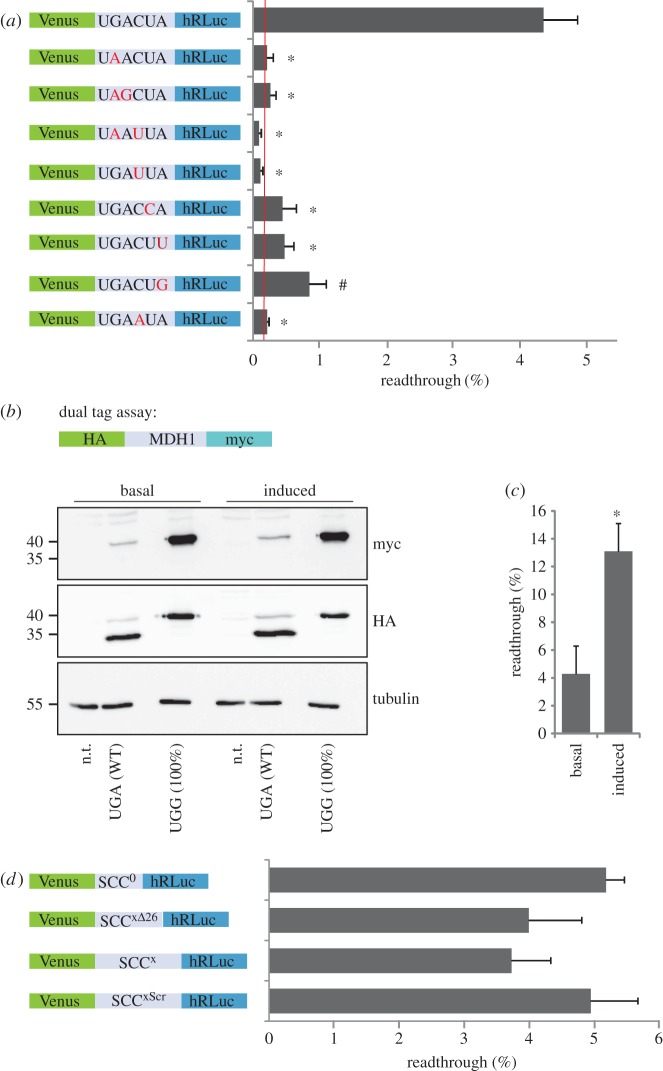
Translational readthrough of human malate dehydrogenase. (*a*) The SCC of MDH1 promotes a high level of translational readthrough. Venus/hRluc dual reporter assay with MDH1 wild-type and mutant SCCs in HeLa cells. Red line indicates background level of readthrough as observed with a construct containing two consecutive UAA stop codons separating Venus and luciferase tags. Mutations are indicated in red. Alterations of the SCC dramatically reduce readthrough efficiency. *N* = 3; *p < 0.01, ^#^*p* < 0.05 versus WT (UGA CUA) (Student's *t*-test). (*b*) Full-length MDH1 is extended by readthrough. Geneticin (100 µg ml^−1^) induces MDH1 readthrough. Western blot of MDH1x (UGA) or MDH1x-UGG (stop codon replaced by Trp codon UGG) containing an N-terminal HA- and a C-terminal myc-tag. Molecular mass marker in kilodaltons; n.t., not transfected. (*c*) Quantification of (*b*). MDH1x readthrough is 4.3 ± 0.82%, treatment with geneticin induced readthrough to 13.1 ± 1.17% (ImageJ, *N* = 3, *p = 0.002). (*d*) Dual reporter assay with MDH1x wild-type SCC (SCC^0^) and MDH1x SCC containing the complete (SCC^x^) or 31/57 nucleotides (SCC^xΔ26^, deletion of the last 26 nucleotides) of the extension. Readthrough does not differ significantly between the constructs, suggesting that the SCC is the main contributor to MDH1x readthrough. SCC^xScr^, MDH1 SCC with a scrambled sequence of the 50 nucleotides following the SCC. *N* = 4. Error bars, s.e.m.

*In silico* modelling of translational readthrough of SCCs based on experimental readthrough data revealed a consensus motif for the SCCs of genes containing high RTP values [15]. MDH1 was the protein with the highest RTP score in this model (electronic supplementary material, figure S1). Ribosome profiling and genome searches for readthrough genes also identified *MDH1* [8,14,16]. To analyse the translational readthrough in detail, we expressed the SCC of *MDH1* comprising 10 nucleotides upstream and downstream of the stop codon in a dual reporter containing N-terminal Venus and C-terminal humanized *Renilla* luciferase (hRLuc) tags in HeLa cells. Luminescence indicated readthrough of the SCCs, and Venus fluorescence served as an internal expression control. As positive controls we used a vector without any SCC (pDRVL) or the SCCs with the tryptophan-coding TGG instead of the TGA stop codon. The background readthrough level was derived from a construct containing two consecutive stop codons separating the Venus and luciferase tags (figure 1*a*) [15]. Readthrough was expressed as hRLuc luminescence divided by Venus fluorescence (in arbitrary units) and ratios were normalized to the 100% controls. Readthrough of the MDH1 SCC was 4.34 ± 0.51% (figure 1*a*). To analyse the influence of the SCC on readthrough, we mutated the stop codon and/or changed the trinucleotide sequence following the stop codon (position +4 to +6). Mutation of the stop codon itself or the C in position +4 of the SCC (UGA CUA, position +4 underlined) reduced readthrough significantly to background level. Mutation of the nucleotides in position +5 or +6 (UGA CUA, positions +5 and +6 underlined) also significantly decreased readthrough compared with the wild-type SCC, but still showed levels between 0.4 and 0.8% (figure 1*a*). In conclusion, the readthrough motif confers high readthrough to the MDH1 SCC and all tested nucleotide changes in the consensus UGA CUA (stop codon underlined) lead to a significant decrease in readthrough.

Readthrough of the (first) MDH1 stop codon is expected to give rise to an MDH1 isoform with a 19 amino acid extension, including the amino acid encoded by the stop codon. We term this isoform MDH1x for extended.

We now turn from the analysis of the SCC to the full-length MDH1. To confirm the extension of the protein by stop codon suppression, we expressed full-length MDH1 including the 57 nucleotides following the stop codon in a dual-tagged vector containing an N-terminal HA-tag and a C-terminal myc-tag replacing the second stop codon (figure 1*b*). Expression was analysed by western blotting and showed 4.3 ± 0.82% full-length MDH1x (figure 1*c*), which is in agreement with readthrough measured for the MDH1 dual reporter construct. Readthrough of the MDH1 stop codon was inducible by the aminoglycoside geneticin (G418) to 13.1 ± 1.17% (figure 1*c*), reliably confirming that the detected signal is consistent with readthrough.

The correspondence of the readthrough level measured for the SCC and the stop codon of the full-length protein suggests that the SCC is the main contributor to stop codon readthrough of MDH1. To analyse the possible influence of the nucleotides following the MDH1 SCC or of mRNA secondary structures that are formed by this sequence on readthrough in more detail, we expressed the MDH1 SCC together with the full downstream stretch of 57 nucleotides encoding the PTS1 (SCC^x^, full extension), the SCC with only 18 nucleotides following the stop codon (SCC^xΔ26^, deletion of 26 nucleotides upstream of the second stop), or the SCC alone (SCC^0^) in a dual reporter experiment. In addition, we expressed the MDH1 SCC together with a scrambled sequence of the 50 nucleotides following the SCC (SCC^xScr^). The scrambled sequence maintains the original length of the extension but would not form the same secondary structure. Measurement of reporter activity shows that readthrough efficiency is not affected by the partial, the complete, or the scrambled nucleotide sequence following the SCC (figure 1*c*). RNA structure predictions of the MDH1 SCC and the readthrough extension did not reveal conserved structures (electronic supplementary material, figure S2). Hence, we conclude that the 50 nucleotides downstream of the MDH1 SCC appear to be dispensable for the modulation of readthrough of the MDH1 stop codon; readthrough of the MDH1 stop codon depends mostly on the SCC. It cannot be excluded, however, that sequence elements downstream of the second stop codon contribute to translational readthrough. Indeed, secondary structure prediction of aligned mammalian MDH1 3′UTRs revealed predicted hairpin structures that may influence readthrough (electronic supplementary material, figure S2*c*). These structural elements are conserved in mammals (electronic supplementary material, figure S3) but they were not found to be conserved in the alignment of vertebrate MDH1 3′UTRs.

### 2.2. Cell type specificity of MDH1 readthrough

**Figure 2.**
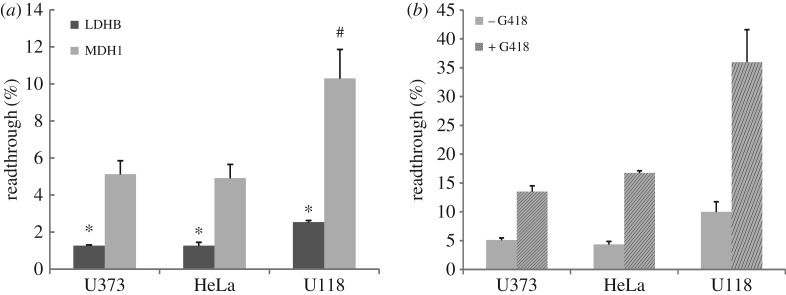
MDH1 readthrough in several cell types and in comparison to LDHB. (*a*) MDH1 stop codon readthrough in various mammalian cell lines. HeLa, U373 and U118 cells were transfected with MDH1 and LDHB SCC reporter constructs and analysed by dual reporter assays. Readthrough is expressed as hRLuc/Venus signal. MDH1 readthrough is significantly higher compared with LDHB readthrough in all cell lines (*p = 0.001 (U373), *p* = 0.002 (HeLa), *p* = 0.001 (U118); Student's *t*-test). MDH1 and LDHB readthrough are highest in U118 cells. MDH1: ^#^*p* = 0.01 (U118 versus HeLa), *p* = 0.01 (U118 versus U373). LDHB: *p* = 3 × 10^−7^ (U118 versus HeLa), *p* = 8 × 10^−5^ (U118 versus U373); Student's *t*-test; *N* = 5. (*b*) Geneticin (100 µg ml^−1^) induces MDH1 readthrough in U373, HeLa and U118 cells. *N* = 3. MDH1 versus LDHB: *p* = 0.002 (U373), 4 × 10^−6^ (HeLa) and 0.01 (U118); Student's *t*-test. Error bars, s.e.m.

When analysing the LDHB SCC in HeLa, HEK, COS-7 and U118 cells using our reporter assay, readthrough was found to be highest in the glioblastoma cell line U118 [15]. We therefore were curious to find out whether MDH1 readthrough would differ in different cell lines. We performed the dual reporter assay in HeLa cells and two glioblastoma cell lines, U118 and U373, and compared MDH1 and LDHB readthrough. Readthrough of the MDH1 SCC was 4.91 ± 0.74% in U373 cells and reached 10.3 ± 3.8% in U118 cells (figure 2*a*,*b*). In all tested cell lines readthrough of the MDH1 SCC was more than twice the level of the LDHB SCC, and both SCCs showed the highest readthrough in U118 cells (figure 2*a*). As before, readthrough was inducible by geneticin. Induction factors ranged between 2.6 in U373 and 3.7 in HeLa cells (figure 2*b*). Under geneticin treatment readthrough of the MDH1 SCC rose to 36 ± 10% in U118 cells (figure 2*b*). These results indicate that readthrough is dependent on and possibly differentially regulated in different cell types.

### 2.3. The MDH1 stop codon encodes tryptophan and arginine

**Figure 3.**
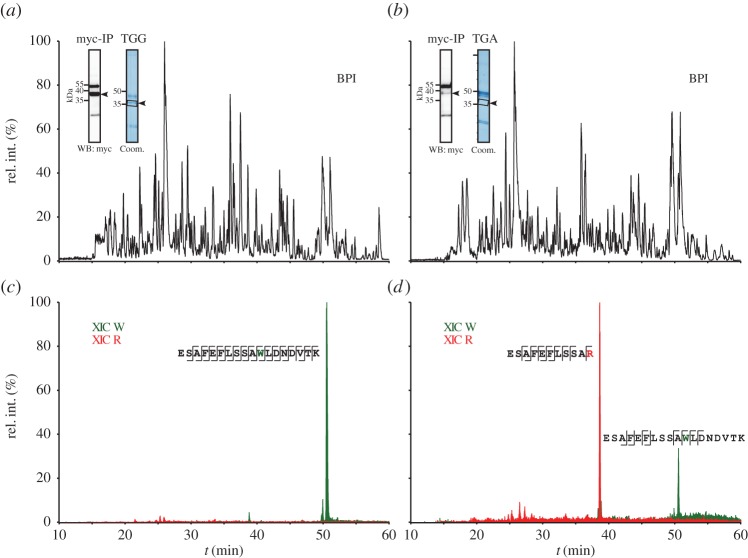
Tryptophan and arginine are incorporated during readthrough at the MDH1 stop codon. (*a*,*b*) LC-MS analysis (base peak intensity chromatogram, BPI) of the tryptic peptides derived from the gel regions that are marked by rectangles in the colloidal Coomassie-stained gel lane shown in the inset. WB: specific detection of myc-tagged proteins by western blotting used to identify gel regions of interest. Intense bands at an apparent molecular weight of approximately 50 kDa and approximately 20 kDa correspond to antibody heavy and light chains, respectively. (*c*,*d*) Mass-selective chromatographic display of the readthrough-related tryptic peptides. Extracted ion chromatograms (XIC) show the peptides resulting from incorporation of Trp (triply charged molecular ion, green trace) or of Arg (doubly charged molecular ion, red trace). Analysis of the MDH1x-derived proteins (*d*) not only confirmed the presence of Trp as seen in the control scenario with Trp-coding (*c*), but additionally revealed the incorporation of Arg that is undetectable in the control. Signal intensities of the two peptides do not reflect the ratio of incorporation of Trp and Arg, respectively. The generated peptides considerably differ in their ionization behaviour, which is particularly due to the emergence of an additional tryptic cleavage site upon incorporation of Arg. For generation of XIC, a mass tolerance window of 20 ppm was applied to continuum data without lock mass correction. Major signals are labelled with the corresponding amino acid sequence together with the fragment ions detected by mass spectrometric sequencing. For the sake of clarity, only C-terminal y-ions and N-terminal b-ions are depicted and neutral loss of ammonia or water is not considered.

Which amino acids are incorporated during readthrough when the stop codon is at the A (aminoacyl-tRNA) site of the ribosome? To answer this question, we expressed C-terminally myc-tagged MDH1x in HeLa cells and immunoprecipitated the fusion protein using anti-myc antibodies. The corresponding construct with the sense codon TGG was used as a control representing full readthrough with incorporation of tryptophan. Precipitated proteins were detected initially by western blotting with anti-myc antibodies (insets in figure 3*a*,*b*). Regions of interest were excised from a gel stained with colloidal Coomassie and subjected to mass spectrometric protein identification (figure 3*a*,*b* and insets). The tryptophan-containing tryptic peptide corresponding to the readthrough region was readily identified by liquid chromatography coupled to mass spectrometry (LC-MS) in the case of the control construct (figure 3*a*,*c*). When analysing the MDH1x proteins, we also detected tryptophan and additionally found arginine, cysteine, glutamine and phenylalanine (electronic supplementary material, table S1). Next, we applied a more stringent criterion for mass spectrometric evidence: the detection of the intact precursor peptide with a high mass accuracy of 3 ppm relative deviation in coincidence with fragment ion series covering the readthrough position. Using this filter, we confirmed the incorporation of tryptophan and arginine in place of the stop codon (figure 3*b*,*d*; electronic supplementary material, table S1). These data suggest that in the endogenous readthrough of the human MDH1 stop codon, the stop codon can encode tryptophan and arginine.

### 2.4. Conserved functional translational readthrough of malate dehydrogenase

**Figure 4.**
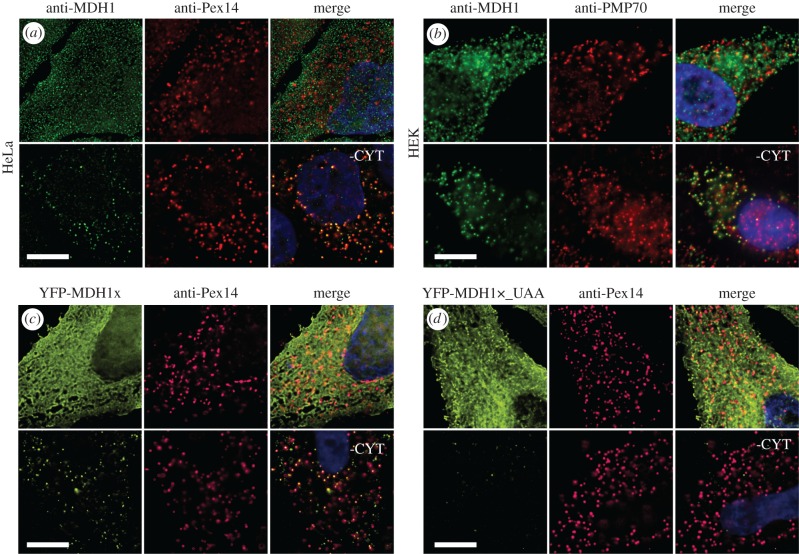
Functional translational readthrough of MDH1. Immunofluorescence with anti-MDH1 and anti-Pex14 in untransfected HeLa (*a*) and HEK (*b*) cells. Endogenous MDH1 shows mainly cytosolic localization. Removal of cytosol (-CYT) after digitonin treatment reveals colocalization of MDH1 with the peroxisomal markers Pex14 and PMP70. (*c*) Peroxisomal targeting of MDH1x depends on the stop codon. Direct immunofluorescence microscopy of transfected HeLa cells: MDH1 localizes mainly to the cytosol. Removal of cytosol (-CYT) after digitonin permeabilization reveals peroxisomal localization of MDH1. (*d*) Exchange of UGA with the tighter stop codon UAA strongly reduces the amount of MDH1x in the peroxisome pre and post removal of cytosol (-CYT). Scale bars, 10 µm.

In our previous study, we searched for transcripts that combined both a high RTP and a high probability of encoding a PTS1 in the readthrough extension [15]. *In silico* analysis of all SCCs of the human genome revealed a high probability of a hidden PTS1 in the readthrough extension of MDH1x. The distribution of RTP^+^ (positively scaled and normalized RTP) × PTS1 product scores over all human transcripts indicates that MDH1x is one of the transcripts containing high RTP and high PTS1 probability (electronic supplementary material, figure S4). Furthermore, MDH1x is targeted to the peroxisome via translational readthrough resulting in expression of the hidden targeting signal [16]. To analyse the endogenous peroxisomal localization of MDH1, we stained endogenous MDH1 and the peroxisomal markers Pex14 or PMP70 in HeLa and HEK cells. As expected, we found that MDH1 is mainly localized in the cytoplasm (figure 4*a*,*b*). To identify the peroxisomal MDH1, we washed out the cytosol with phosphate buffered saline (PBS) after cell permeabilization by digitonin. After cytosol removal, endogenous MDH1 was readily detected in peroxisomes (figure 4*a*,*b*). To be able to test whether the hidden targeting signal in the MDH1x extension is responsible for peroxisome targeting, we transfected HeLa cells with YFP-tagged MDH1x. The fusion protein could be detected in the peroxisomes after removal of the cytosol (figure 4*c*). To prove that targeting of MDH1x to the peroxisomes is dependent on the type of the stop codon, we mutated the stop codon UGA to the more efficient stop codon UAA. This tighter stop codon reduced the peroxisomal targeting of MDH1x (figure 4*d*). When the stop codon was mutated to a sense codon (UGG, tryptophan-coding), peroxisomal localization of MDH1x was strongly increased and obvious even without washing out the cytosol (electronic supplementary material, figure S5*a*). To show that peroxisomal targeting of MDH1x is dependent on the hidden PTS1 in the extension, we mutated the PTS1 in the extension by deletion of the leucine residue (ΔL) in the CRL-terminus. This mutation blocks import of MDH1x into peroxisomes (electronic supplementary material, figure S5*b*), indicating that expression of the PTS1 in the extension is necessary for peroxisomal targeting of MDH1x.

Furthermore, to test whether functional readthrough of the MDH1 stop codon is a general property, we stained endogenous MDH1 in two glioblastoma cell lines, U118 and U373, and in murine cardiomyocytes. Immunofluorescence with antibodies directed against MDH1 and the peroxisomal membrane proteins PMP70 and Pex14 showed mainly cytoplasmic localization of the endogenous MDH1 (electronic supplementary material, figure S6*a*). After removal of the cytosol, we found that MDH1 is localized to peroxisomes in all cell types tested (electronic supplementary material, figure S6*a*). In cardiomyocytes, endogenous MDH1 is already detectable without removal of cytosol (electronic supplementary material, figure S6*b*). This indicates that the readthrough-extended isoform of MDH1 is imported into peroxisomes in various cell types and further substantiates the evolutionary conservation of peroxisomal requirement of MDH1.

**Figure 5.**
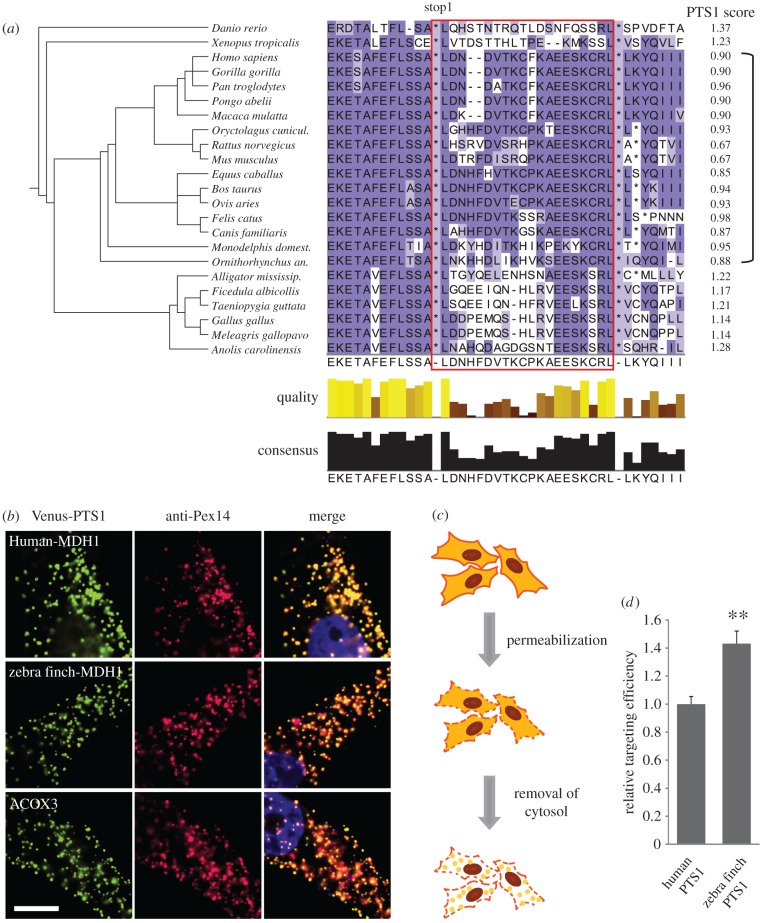
Assessing the ‘bird gap’: comparative analysis of the zebra finch and human hidden PTS1. (*a*) Alignment of MDH1x termini in mammals and non-mammalian vertebrates. MDH1x is conserved throughout vertebrates. Red box marks readthrough extension. Bracket on the right-hand side: Mammals. PTS1 score calculation suggests that mammalian MDH1x PTS1 is weaker than the PTS1 of non-mammalian vertebrate MDH1x. Non-mammalian species, however, do not have LDHBx (bird gap). (*b*) PTS1 of both human and zebra finch (*Taeniopygia guttata*) MDH1x localize to peroxisomes. PTS1 from peroxisomal membrane protein ACOX3 was used as a positive control. Scale bar, 10 µm. (*c*) Quantitative live analysis of peroxisomal protein import efficiency. Venus-tagged PTS1 was expressed in HeLa cells. After digitonin permeabilization of the plasma membrane, the cytoplasm was washed out by PBS. Hundred cells from images pre and post removal of cytoplasm were used to calculate import efficiency. (*d*) Analysis of Venus fluorescence before and after removal of cytoplasm revealed that targeting efficiency of zebra finch Venus-tagged MDH1x PTS1 is more efficient compared with human MDH1x PTS1. **p = 0.005 versus human PTS1 (Student's *t*-test), *N* = 5. Error bars, s.e.m.

To assess whether MDH1 readthrough and the hidden PTS1 are a common feature in mammals and vertebrates, we performed a phylogenetic analysis on MDH1 transcript sequences of a wide range of vertebrate species. The multiple alignment of potential readthrough extensions of MDH1x orthologues in mammalian and non-mammalian vertebrates illustrates that the extension including the hidden PTS1 is conserved among these species (figure 5*a*), supporting the notion that the hidden PTS1 is functional in all vertebrates. All mammalian MDH1x PTS1 contain the terminal tripeptide CRL, and all non-mammalian PTS1 contain SRL.

Next, we calculated PTS1 prediction scores for the MDH1x proteins of the alignment (figure 5*a*). We found that the non-mammalian signal (SRL) is predicted to be the more efficient PTS1 when compared with the hidden PTS1 found in mammalian MDH1x (CRL) (figure 5*a*). This coincides with the exclusive presence of LDHBx in mammals.

To prove the functionality of these putative PTS1 motifs, we transfected HeLa cells with a Venus-tagged construct expressing the last 10 amino acids from the human MDH1x (terminating tripeptide CRL) and from zebra finch (*Taeniopygia guttata*) MDH1x (terminating tripeptide SRL). The Venus-tagged PTS1 derived from the peroxisomal matrix protein ACOX3 was used as a positive control. Cells were co-stained with anti-Pex14 antibody. Both human and zebra finch Venus-MDH1-PTS1 localize to peroxisomes in HeLa cells (figure 5*b*). This indicates that both zebra finch and human MDH1x PTS1 are functional and that both SRL and CRL are good targeting signals for peroxisomal import. To evaluate the differences in mammalian and non-mammalian MDH1x peroxisomal targeting, we compared the targeting efficiencies of zebra finch and human PTS1 quantitatively. To analyse the relative targeting efficiency fluorescence signals of the cells before and after removal of cytosol were analysed and the ratio of peroxisomal versus total signal of the PTS1 constructs was calculated. We found that 43% more of zebra finch MDH1x PTS1 was localized to the peroxisomes compared with the human MDH1x PTS1. This indicates that the zebra finch PTS1 terminating in SRL is a more efficient targeting signal than the human CRL PTS1 (figure 5*d*).

## 3. Discussion

### 3.1. Functional translational readthrough generates MDH1x

Translational readthrough has only recently been identified and systematically analysed in mammals [17]. Three systems biology approaches, ribosome profiling, phylogenetic analysis, and regression modelling, are being used to search for new protein isoforms arising from stop codon readthrough [17]. MDH1x is the only protein that has been identified by all three approaches [8,14–17]. In this and our previous study on LDHBx, we used the SCC regression model to characterize endogenous stop codon readthrough [15]. The SCC motif [14–16] responsible for high readthrough levels is found in 57 human translational readthrough candidates. Of those, MDH1 has the highest RTP.

Readthrough of the MDH1 stop codon is dependent on the SCC motif UGA CUA (stop codon underlined). At this point, there is no general agreement whether readthrough is mainly influenced by the SCC or whether *cis*-acting mRNA elements downstream of the stop codon promote readthrough. We addressed this question by using a quantitative assay that enabled us to compare readthrough levels of different SCCs, and to analyse readthrough in different cell lines. We show here that the mRNA sequence downstream of the *MDH1* SCC is not required to obtain the 4% readthrough that is already sponsored by the SCC alone. The predicted mRNA structure of the MDH1 extension is not conserved, which is in agreement with our finding that translational readthrough of MDH1 is not dependent on the full extension but rather on the SCC alone. Nevertheless, these findings do not preclude a role of the extension in general, such as for RNA stability, or a contribution of the mRNA elements downstream of the second stop codon.

MDH1 readthrough was present in all cell types analysed in our study, and was found to be highest in U118 glioblastoma cells. The different readthrough properties of these cell lines could be due to different levels of metabolic dysregulation following the oncogenic transformation of these cells that could manifest in different tRNA or release factor concentrations.

In agreement with the higher RTP value of MDH1, readthrough intensity of MDH1 exceeded LDHB readthrough more than twofold in all tissues tested. LDHB readthrough was also highest in U118 cells, suggesting an overall increase in readthrough probability in these cells that is possibly due to the different metabolic regulation.

MDH1 is also known to contain a functional hidden PTS1 in the extension [16] and endogenous MDH1 is present in the peroxisomes of HeLa cells, HEK cells, glioblastoma cell lines and murine cardiomyocytes.

### 3.2. Translational readthrough constitutes a modification of the genetic code in humans

The fidelity of amino acid incorporation at the ribosome is controlled by selection of the tRNA and by kinetic proofreading after GTP hydrolysis by the elongation factor [43]. Compared with cognate and near-cognate tRNAs, non-cognate tRNAs are not able to induce GTP hydrolysis. Structural analysis of ribosomes revealed that the shape of the base pairs, and not numbers or types of hydrogen bonds, determines tRNA selection [44], and that ‘geometrical mimicry’ by non-standard base pairings can lead to incorporation of near-cognate tRNAs [45].

In this study, we show amino acid incorporation during endogenous readthrough of a human protein in the absence of pharmacological induction or other readthrough-enhancing conditions. Mass spectrometric analysis provided evidence of predominant incorporation of tryptophan (codon UGG) and arginine (codons CGX, AGR; X = A,G,C, or U; R = A or G) at the UGA stop codon of human MDH1x. This finding suggests that readthrough of stop codons results from stop codon recognition by near-cognate tRNAs, as they can form at least two out of the three pairings with the stop codon. Our results support the observation that the second position of the stop codon is crucial for acceptance of a tRNA at the A-site of the ribosome [46], and that mispairing occurs at the first or third position of the stop codon.

The first systematic mass spectrometric analysis of the amino acids incorporated at stop codons was undertaken recently in *Saccharomyces cerevisiae* using the high readthrough strain [PSI^+^] [46]. Glutamine, lysine and tyrosine were found to be incorporated at UAA and UAG stop codons, and tryptophan, arginine and cysteine at the UGA stop codon. Endogenous readthrough of premature termination codons in yeast leads to insertion of tryptophan, arginine and cysteine [47]. These results in yeast agree with the identification of the corresponding suppressor tRNAs using *in vitro* expression systems or *E. coli* infected with RNA phage Qβ [3,5,48–50].

Using an *in vitro* expression system, mass spectrometric analysis of amino acid incorporation in rabbit β-globin also found tryptophan, arginine, cysteine and, additionally, serine at the UGA stop codon [11]. *In vitro* expression of the murine leukemia virus *pol* gene leads to formation of a fusion protein due to readthrough of the UGA stop codon. During this process, tryptophan, arginine and cysteine are integrated at this stop codon [51].

Whether the other nucleotides of the SCC influence amino acid incorporation during the readthrough process is still not known. On the one hand, it was suggested that the surrounding nucleotides have no influence on amino acid incorporation [46], but another study in yeast shows that the UGA stop codon followed by A is preferentially readthrough by Trp-tRNA, whereas it is readthrough by Cys-tRNAs when followed by G or C [52].

Moreover, it would be helpful to determine whether the amino acid profile changes when readthrough is induced pharmacologically. Recent work in yeast indicates that the same amino acids are incorporated at the UGA stop codon, but with different insertion frequencies [47].

### 3.3. Potential roles of a peroxisomal malate dehydrogenase

Malate dehydrogenase is universally present in peroxisomes and employs a surprising variety of targeting strategies: plants contain two peroxisome-specific *MDH* genes which are targeted to the peroxisome via PTS type 2 [53–57]. *Saccharomyces cerevisiae* has three MDH isoforms: mitochondrial MDH1, cytoplasmatic MDH2 and peroxisomal MDH3. The latter uses a dedicated PTS1 [58–63]. In *Yarrowia lipolytica*, a peroxisome-specific MDH isoform results from alternative splicing, creating an mRNA containing a PTS1 [64]. Proteomic analyses additionally found MDH1 in mammalian peroxisomes [41,42]. Taken together, these findings suggest the evolutionary conservation of MDH1 requirement in peroxisomes.

In *S. cerevisiae*, peroxisomal β-oxidation is blocked when the *MDH3* gene is disrupted [59]. Peroxisomal β-oxidation of fatty acids requires a peroxisomal pool of NAD^+^, which is reduced to NADH in this process. However, neither NAD^+^ nor NADH are able to cross the peroxisomal membrane [59,65,66]. NAD^+^, therefore, has to be recycled from NADH within the peroxisome. As MDH catalyses the conversion of oxaloacetate and NADH to malate and NAD^+^, it was hypothesized that MDH3 is involved in this process [59]. The identification of MDH1x provides a mechanistic explanation for MDH1 import into peroxisomes of vertebrate cells and suggests that in all cell types, MDH is potentially involved in regeneration of redox equivalents in the peroxisomal matrix.

The universal presence of MDH in peroxisomes may suggest a malate shuttle across the peroxisomal membrane of vertebrate cells resembling the aspartate–malate shuttle of the mitochondrial membrane that was first identified in rat heart [67,68]. Like the peroxisomal membrane, the mitochondrial membrane is impermeable to NAD^+^ and NADH [69] and, therefore, NADH has to be generated by mitochondrial MDH. The oxoglutarate : malate antiporter imports malate into the mitochondrial matrix where it is converted to oxaloacetate. Oxaloacetate is then converted to aspartate and shuttled back to the cytoplasm where oxaloacetate is regenerated, which is the substrate of the cytoplasmic MDH. Strikingly, in yeast the cytoplasmic MDH2 is required for peroxisome function [70] lending support to our hypothesis of a universal peroxisomal malate shuttle.

Mitochondrial and cytoplasmic aspartate aminotransferases catalyse the interconversion of aspartate and oxaloacetate. Remarkably, this protein was also found in peroxisomes of *S. cerevisiae* and various plant species [71–73], which supports the hypothesis of a malate/aspartate shuttle across the peroxisomal membrane. However, disruption of the peroxisomal aspartate aminotransferase in *S. cerevisiae* did not lead to a β-oxidation defect, and mutants were able to grow normally on oleate, thus indicating that the peroxisomal aspartate aminotransferase is not essential for peroxisomal function [73].

### 3.4. The antiquity of peroxisomal malate dehydrogenase

LDHB can be readthrough-extended to form LDHBx and is transported into the peroxisome by an otherwise hidden PTS1 [15]. The non-extended subunits of LDH, LDHA and LDHB, can be transported into the peroxisome via piggyback transport so that LDH tetramers are present inside peroxisomes [15,38]. The LDHBx extension including the hidden targeting signal is conserved in mammals but it is not present in non-mammalian species (bird gap). On the other hand, the MDH1x extension and the hidden PTS1 are conserved in mammalian and non-mammalian vertebrates, indicating evolutionarily conserved peroxisomal targeting. Of note, all mammalian MDH1x encode a PTS1 terminating with the tripeptide CRL. The PTS1 tripeptide of the non-mammalian vertebrates (excluding amphibia) is SRL, which is predicted to be a more efficient PTS1 and we proved that PTS1 targeting of zebra finch PTS1 (SRL) is more efficient than targeting of human PTS1 (CRL). We therefore suggest that peroxisomal import of all non-mammalian vertebrate MDH1x is more efficient compared with the MDH1x import in mammals. Mammals, however, (also) have LDHBx. We further hypothesize that the role which is shared between peroxisomal LDH and MDH1x in mammals is carried out by MDH1x alone in non-mammalian vertebrates. It is plausible that the evolution of LDHBx in the mammalian clade was the precondition for the weakening of the MDH1x PTS1.

LDHB and MDH1 are closely related, with MDH being the evolutionarily older enzyme [74]. The amino acid sequence of the halobacterial MDH, for example, shows greater sequence similarity to other LDHs than to other MDHs [75], and its X-ray structure is more similar to LDH than to other MDHs [76]. Exchange of only one amino acid can convert bacterial LDH substrate specificity from lactate to malate [77]. MDH substrate specificity can also be changed from oxaloacetate to pyruvate by one amino acid change [75]. These findings underline the close relation of both enzymes and thus support the hypothesis that both exert similar functions in the peroxisome. In the light of the common ancestry of MDH and LDH, it is intriguing that both enzymes have adopted a modification of the genetic code and harness the coding potential of the 3′ end of their transcripts. *Functional translational readthrough*, therefore, expands the human proteome and the options of its intracellular targeting.

## 4. Material and methods

### 4.1. DNA cloning

Plasmids used in this study are listed in the electronic supplementary material, table S2. Oligonucleotides used are listed in the electronic supplementary material, table S3. Dual reporter constructs were cloned based on pDRVL (PST1360) encoding an N-terminal Venus tag and a C-terminal hRluc tag [15]. For dual reporter constructs PST1521, 1523, 1581–82, 1525–26, 1473, 1474, 1475, 1476, 1479, 1480, 1481 and 1502, oligonucleotides JH 77–78, JH 105, 106, 109, 110, 113, 114, 137–140, 161–164 and OST1463–1470 and 1475–1480 were annealed and inserted into BspEI and BstEII sites of pDRVL. For cloning of pEYFP-MDH1x (PST1436) the MDH1 open reading frame including the stop codon and the 57 nucleotide 3′ extension was PCR-amplified from human cDNA using primers OST1192 and 1193 and inserted into EcoRI and BamHI sites of pEYFP-C1. The stop codon variants pEYFP-MDH1xTGG (PST1514) and pEYFP-MDH1xTAA (PST1535) were created by amplifying MDH1x from PST1436 using primers OST1231 and 1232, and JH103 and 104. Similarly, the deletion of the last amino acid in the cryptic PTS1 CRL, pEYFP-MDH1TGGxΔL (PST1536 deletion of the last amino acid in the cryptic PTS1 CRL) was created from PST1514 using primers OST1192 and JH102. Full-length dual reporter construct pcDNA3.1-HA-MDH1x-myc (PST1443) was cloned by amplification of MDH1x from PST1436 with primers OST1204 and 1205 and insertion into NheI and BamHI sites of pcDNA3.1/Myc-His(−)A. The stop codon mutant pcDNA3.1-HA-MDH1TGG-myc (PST1508) was generated by site-directed mutagenesis using primers OST1231 and 1232. pEXP-N-Venus-PTS1-human (PST1482) and pEXP-N-Venus-PTS1-zebra-finch (1483) were cloned using Gateway technology (Invitrogen). After annealing of primers OST1245 and 1246, and OST1247 and 1248, they were inserted into the entry vector pENTR/D-TOPO by TOPO-D cloning reaction. Inserts were then transferred to pEXP-N-Venus by site-specific recombination using the LR reaction. All plasmids were confirmed by DNA sequencing.

### 4.2. Cell culture

HeLa cells were maintained in low glucose Dulbecco's minimal essential medium (DMEM), and HEK, U118 and U373 cells in high glucose DMEM. Culture media were supplemented with 1% glutamine, 5–10% heat-inactivated fetal calf serum (FCS), 100 units ml^−1^ penicillin and 100 µg ml^−1^ streptomycin. For U118 and U373 cells, 1% non-essential amino acids and 1% pyruvate were added to the media. Cells were transfected using Effectene transfection reagent (Qiagen) as described by the manufacturer. Six hours after transfection, transfection reagent was removed and, as indicated, 100 µg ml^−1^ geneticin (G418) was added.

### 4.3. Dual reporter assays and readthrough calculation

For dual reporter assay, approximately 6500 cells were seeded per well in a 96-well plate and transfected as indicated; 24 h after transfection Venus fluorescence and hRluc luminescence were measured by the Renilla Luciferase Assay System (Promega) and the Synergy Mx plate reader (Biotek). Cells were washed with PBS and lysed in 30 µl Renilla Luciferase Assay Lysis Buffer (Promega) according to the manufacturer's manual. Cells were loosened from the surface using a pipette tip and were incubated on a shaker for 15 min at room temperature. For fluorescence measurement 50 µl Renilla Luciferase Assay Reagent without substrate was added per well and the lysates were analysed at 485 nm excitation, 530 nm emission (sensitivity: 130) in the plate reader. For hRluc luminescence measurement, 50 µl Assay Reagent containing 2× substrate was added to the lysates using an automated injector. Luminescence was read 2 s after injection and integrated over 10 s (sensitivity 150). Renilla Luciferase Assay Reagent plus PBS was used as a blank control for both measurements. Each construct was analysed in three to seven biological replicates. The readthrough rates were calculated as explained [15]. Briefly, the ratio of hRluc/Venus fluorescence was calculated, and the construct ratios of pDRVL were set to 100%. For measurement of SCC^x^, SCC^xScr^ and SCC^xΔ26^, pDRVL containing the same insert with TGG mutation of the stop codon was used as 100% control. Arithmetic mean and standard error of the mean (s.e.m.) were calculated for each construct from its biological replicates.

### 4.4. Immunofluorescence and microscopy

Transfected MDH1 constructs and endogenous MDH1 were detected in HeLa cells by combined direct/immunofluorescence experiments. Approximately 10^5^ cells were seeded on coverslips and transfected as indicated by Effectene Transfection Reagent as described above. Coverslips were coated with laminin (Sigma) for U118 cells and with poly-l-lysine (Sigma) for HEK cells. For removal of cytosol, cells were treated with 0.02% digitonin (Invitrogen) for 5 min at room temperature. Cells were fixed with 10% formaldehyde for 20 min, and permeabilized using 0.5% Triton X-100 in PBS for 5 min. After blocking for 20 min at 37°C with 10% BSA in PBS, antigens were labelled with primary antibodies at 37°C for 1 h. Primary antibody dilutions in blocking buffer were 1 : 200 rabbit anti-PEX14 (ProteinTech), 1 : 100 mouse anti-MDH1 (Abcam), 1 : 100 rabbit anti-MDH1 rabbit (Sigma) and 1 : 500 mouse anti-PMP70 (Sigma). Secondary labelling was done for 1 h with antibodies (1 : 200) conjugated to Cy3 or Alexa647 (Jackson Immuno Research), or Alexa488 (MoBiTech). Coverslips were mounted with Vectashield mounting medium with DAPI (Vector Laboratories). Isolation of mouse cardiomyocytes was done by Langendorff perfusion and collagenase type II (2 mg ml^−1^, Worthington Biochemical Corporation) digestion. Jonas Peper isolated cardiomyocytes using published protocols [78,79]. Freshly isolated cardiomyocytes were seeded on laminin-coated coverslips for 60 min. Cells were fixed with 4% paraformaldehyde for 10 min and incubated in blocking solution (10% FCS, 0.2% Triton X-100 in PBS) for 1 h. Primary and secondary antibodies were diluted as mentioned above in blocking buffer and incubated overnight at 4°C. Fluorescence microscopy was done using a 100× oil objective (1.3 NA) with a Zeiss Imager M1 fluorescence wide field microscope equipped with a Zeiss AxiocamHRm Camera and Zeiss Axiovision 4.8 acquisition software. z-Stacks with 20 images and 0.2 µm spacing were recorded and subjected to deconvolution. Linear contrast enhancements were applied using Axiovision software.

### 4.5. Cell lysis, western blot analysis

Cells were lysed in RIPA lysis buffer (20 mM Tris–HCl, pH 7.4, 150 mM sodium chloride, 2 mM EDTA, 1% NP40, 1 mM DTT, 0.1 mM PMSF, Complete protease inhibitors (Roche)), 24 h after transfection. Proteins were separated by SDS-PAGE on a 12% gel, transferred to a nitrocellulose membrane and probed with primary and secondary antibodies. The following antibodies were used at a 1 : 1000 dilution: rabbit polyclonal anti-HA (Abcam), mouse monoclonal anti-myc (Cell Signaling), and mouse monoclonal anti-tubulin (Sigma). HRP-conjugated goat anti-rabbit IgG and donkey anti-mouse IgG (Jackson Immuno Research) were used as secondary antibodies at 1 : 5000. Reactive bands were revealed with Lumi-light and Lumi-light plus western blotting substrate (Roche). Luminescence was recorded using luminescent image analyser LAS 4000 (Fuji). Densitometric analysis was done using ImageJ. Readthrough was expressed as ratio of: intensity of MDH1x band (readthrough band)/intensity of MDH1x band + intensity of MDH band.

### 4.6. Immunoprecipitation and mass spectrometric analysis

HeLa cells were seeded in a 10 cm-plate and transfected with C-terminally myc-tagged MDH1x and MDH1TGGx constructs (PST1443 and PST1508); 24 h after transfection, cells were harvested and lysed in 200 µl Cellytic buffer (Sigma) with 1 mM DTT, 1 mM EDTA and Complete protease inhibitors (Roche) for 30 min on ice. After centrifugation, 3 µl anti-myc-antibody (Cell signaling) was added to the supernatant and the mixture was incubated for 1 h at 4°C on a rotating wheel, then 20 µl PBS-washed protein G agarose beads (Thermo Scientific) were added for 3 h at 4°C. Beads were washed 3× with Cellytic and 3× with PBS. Bound proteins were eluted with 4× Roti-Load2 (Roth). IP efficiency was analysed by western blot using 1/4 of the sample, and 3/4 were used for mass spectrometric analysis. For this purpose, proteins were separated on precast TG PRiME Tris/glycine 8–16% gradient gels (Serva) and visualized by colloidal Coomassie staining. Gel regions of interest were excised manually and subjected to automated in-gel digestion with trypsin as described previously [80]. Nanoscale reversed-phase UPLC separation of tryptic peptides (peptides resulting from trypsin digestion) was performed with a nanoAcquity UPLC system equipped with a Symmetry C18 5 μm, 180 μm × 20 mm trap column and a BEH C18 1.7 μm, 75 μm × 100 mm analytical column (Waters Corporation). Peptides were separated over 60 min at a flow rate of 300 nl min^−1^ with a linear gradient of 1–45% mobile phase B (acetonitrile containing 0.1% formic acid) while mobile phase A was water containing 0.1% formic acid. Mass spectrometric analysis of tryptic peptides was performed using a Synapt G2-S quadrupole time-of-flight mass spectrometer equipped with ion mobility option (Waters Corporation). Positive ions in the mass range *m*/*z* 50–2000 were acquired with a typical resolution of at least 20 000 FWHM (full width at half maximum) and data were lock mass corrected post-acquisition. Data acquisition and processing was performed as described [81]. For identification of the myc-tagged MDH1 constructs, data were searched against a custom database with 21 entries, which was compiled on the basis of the construct sequence by introducing each of the proteinogenic amino acids or a one-residue gap in the readthrough position. Precursor and fragment ion mass tolerances were typically below 5 ppm for precursor ions and below 10 ppm (root mean square) for fragment ions. Carbamidomethylation of cysteine and oxidation of methionine were specified as variable modifications. One missed trypsin cleavage was allowed.

### 4.7. *In vivo* measurement of peroxisomal protein import efficiency

HeLa cells were seeded in 6-well plates on coverslips, transfected with Venus-tagged MDH1 targeting signals (last 10 amino acids from zebra finch and human MDH1x) and transferred into a live imaging chamber. Cells were maintained in physiological buffer (in mM: 140 NaCl, 2.5 KCl, 1.8 CaCl_2_, 1.0 MgCl_2_, 20 glucose, 20 HEPES, pH 7.4) and were imaged using a Zeiss Imager M1 fluorescence wide field microscope equipped with a Zeiss AxiocamHRm Camera and Zeiss Axiovision 4.8 acquisition software. The buffer was then exchanged with buffer containing 0.006% digitonin (Sigma) to permeabilize the cells and remove the cytoplasm. Fluorescence was measured each minute for 12 min in total. After 12 min the chamber was washed again with fresh buffer to remove remaining cytoplasmic fluorescence. Mean fluorescence intensity of 100 cells of the 0 min-image and the image after the last wash were measured using ImageJ. After background subtraction, the ratio of fluorescence pre and post digitonin wash was calculated to analyse import efficiency.

### 4.8. Statistics

Statistical analysis was done with Excel using the Student's *t*-test for repeated measurements. Data were presented as means ± s.e.m. (standard error of the mean). *p*-values < 0.05 were considered statistically significant.

## Supplementary Material

Supplementary Information

## Data accessibility

All data supporting this article have been included in the main text, figures and in the electronic supplementary material. The electronic supplementary material comprises six figures (figures S1–S6) and three tables ( tables S1–S3).
