# Supplementary material for: The functional readthrough extension of malate dehydrogenase reveals a modification of the genetic code
Source: Open Biol. 2016 Nov 23;6(11):160246. doi: 10.1098/rsob.160246 (PMC5133446; doi:10.1098/rsob.160246)
Supplement: Supplementary Information [file rsob160246supp1.pdf]

Supplementary Material

The functional readthrough extension of malate dehydrogenase reveals a modification of the genetic code

Julia Hofhuis[1], Fabian Schueren[1], Christopher Nötzel[1,4], Thomas Lingner[2], Jutta Gärtner[1], Olaf Jahn[3], Sven Thoms[1,*]

[1] Department of Pediatrics and Adolescent Medicine, University Medical Center Göttingen, University of Göttingen, 37075 Göttingen, Germany

[2] Microarray and Deep Sequencing Core Facility, University Medical Center Göttingen, University of Göttingen, 37077 Göttingen, Germany

[3] Proteomics Group, Max-Planck-Institute of Experimental Medicine, 37075 Göttingen, Germany

[4] Current Address: Program in Biochemistry and Structural Biology, Cell and Developmental Biology, and Molecular Biology, Weill Cornell Graduate School of Medical Sciences, New York, NY, United States of America

[*] Correpondence: Email: sven.thoms@med.uni-goettingen.de

Supplementary Figures S1 – S6

Supplementary Tables S1 – S3

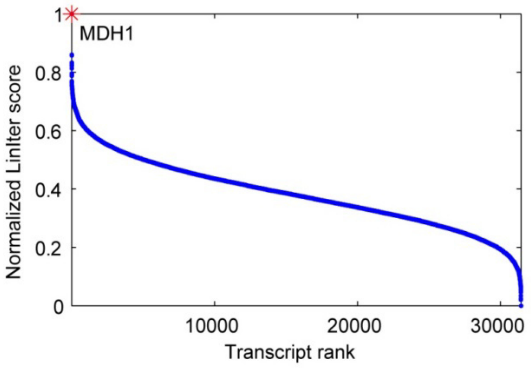

Supplementary Figure S1. Genomic distribution of readthrough propensity. Positively scaled and normalized LinIter scores (RTP$^+$) of the 31,000 unique SCCs are plotted versus the transcript rank. LinIter: regression coefficients for the iterative model considering all 12 stop context positions.

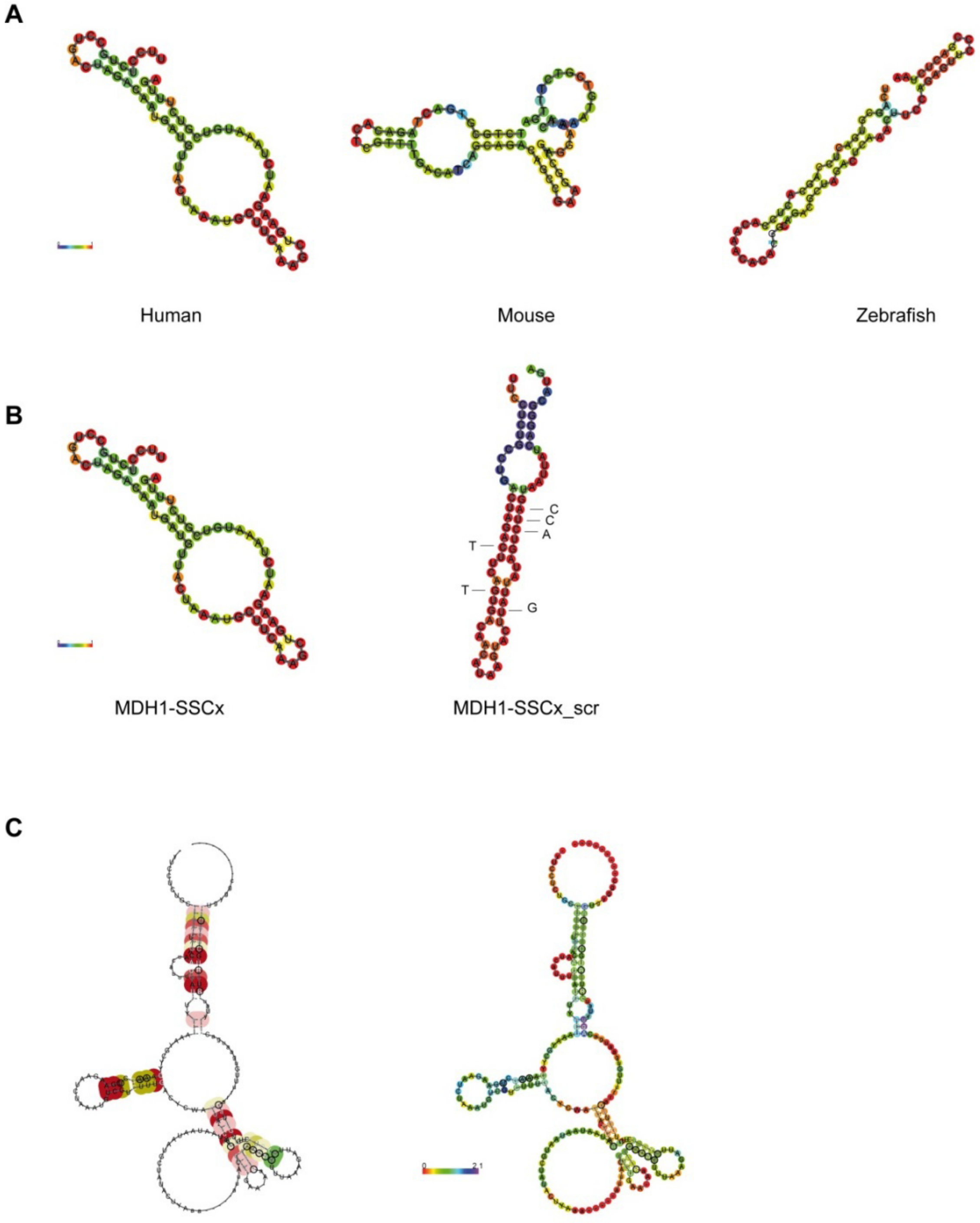

Supplementary Figure S2. Lack of conservation in RNA structural elements in the readthrough extension of vertebrate MDH1x. (**A**) mRNA structure prediction was performed with RNAfold Web Server using nucleotides from position -10 up to the second stop codon. MDH1 extensions shown: Human (*Homo sapiens*), Mouse (*Mus musculus*), and Zebra fish (*Danio rerio*). (**B**) mRNA structure prediction of MDH1-SCCx and MDH1-SCCx with scrambled extension (MDH1-SCCx_scr). (**C**) Conserved predicted secondary structure based on alignment of the mammalian MDH1 3'UTRs (nucleotides -10 up to +180, see also alignment in Supplementary Fig. S3) using RNAlifold Web

Server. Left: structure highlighted conserved elements like in Supplementary Fig. S3, right: base-pair probabilities.

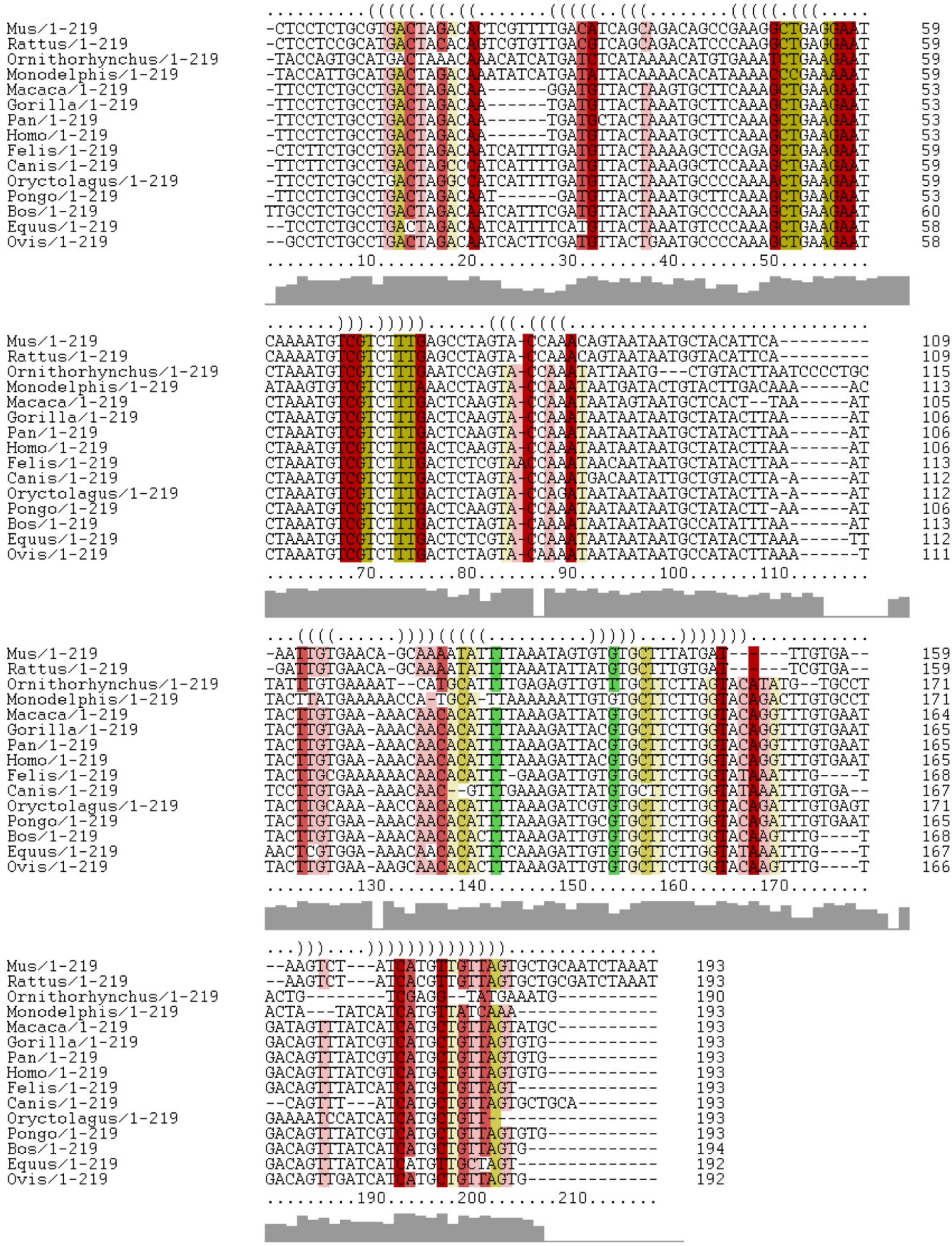

Supplementary Figure S3. Structure annotated alignment of mammalian MDH1 3'UTRs. Mammalian MDH1 3'UTRs (nucleotides -10 to +180) were aligned using Multiple Sequence Alignment Tool Clustal Omega and the alignment was then used for prediction of consensus secondary structures (RNAlifold Web Server). Dot-bracket notation shows the optimal secondary structure with a minimum free energy.

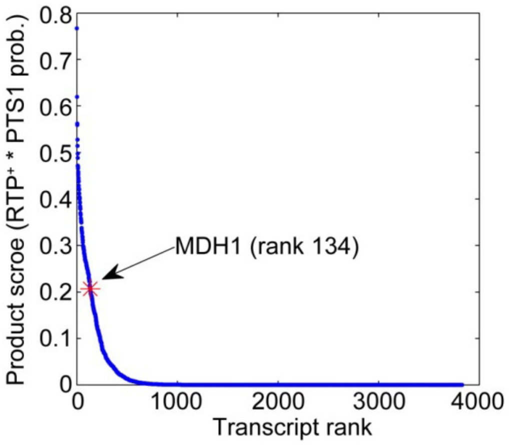

Supplementary Figure S4. Genomic distribution of product score. Product score of scaled and normalized LINiter readthrough propensity scores (RTP^+) and PTS1 targeting probabilities plotted versus the rank of the transcript for the first 4000 out of 31,000 unique SCCs.

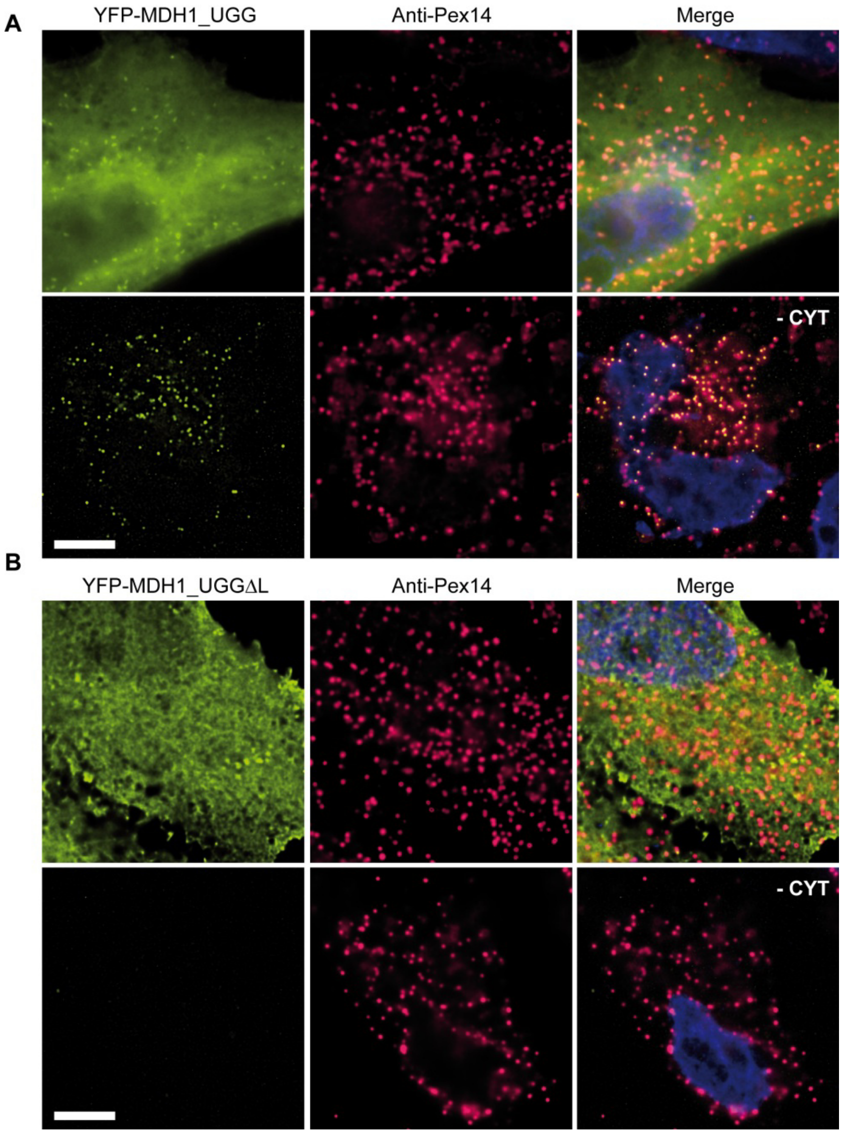

(Supplementary Figure S5)

Supplementary Figure S5. MDH1x targets to the peroxisome by translational readthrough and a hidden peroxisomal targeting signal type1 (PTS1). (**A**) Direct immunofluorescence microscopy of transfected HeLa cells. YFP-MDH1_UGG localizes to the cytoplasm and peroxisomes. Removal of cytosol (- CYT) after digitonin treatment allows visualization of the peroxisomal localization of MDH1. (**B**) Peroxisomal targeting of MDH1 is dependent on the hidden PTS1 CRL in the extension. Deletion of leucine (ΔL) in CRL blocks import of MDH1 into peroxisomes. Scale bar: 10 µm

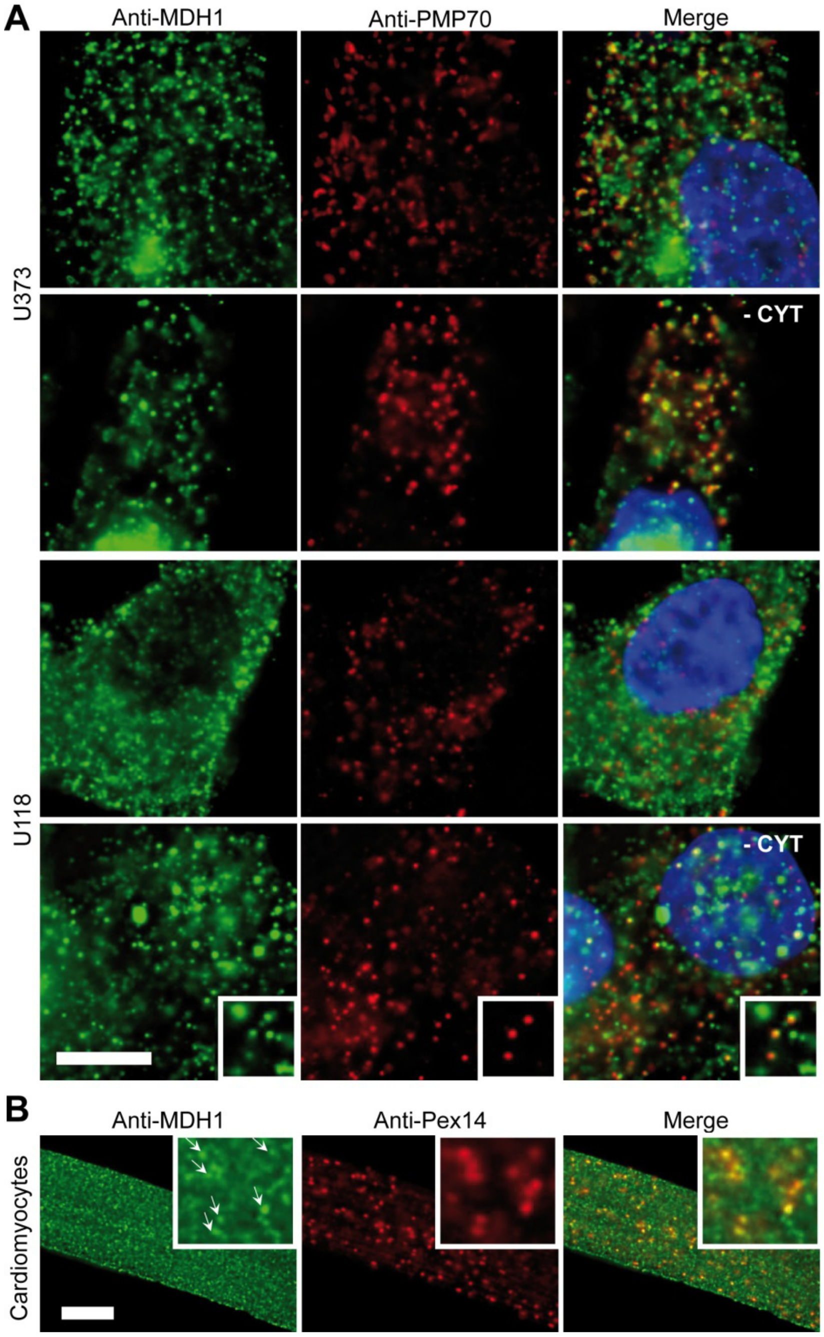

Supplementary Figure S6. Endogenous MDH1 localizes to peroxisomes in glioblastoma cells and murine cardiomyocytes. Immunofluorescence with anti-MDH1 and anti-Pex14 or anti-PMP70 antibodies. (**A**) Endogenous MDH1 shows mainly cytosolic localization in U118 and U373 cells. Detection of colocalization of MDH1 with the peroxisomal marker PMP70 after removal of cytosol (- CYT). (**B**) In murine cardiomyocytes MDH1 colocalizes with Pex14 on top of the cytosolic localization of MDH1. Arrows indicate peroxisomal localization of MDH1. Scale bars: 10 µm.

	Amino acids detected in this study					Amino acids detected by other studies using eukaryotic translation systems		
	Peptide	Intensity [a.u.]	ΔM(rel) [ppm]	b-y	RMS error [ppm]	Feng et al., 1990	Geller and Rich, 1980	Chittum et al., 1998
**Arginine**	(K)ESAFEFLSSAR(L)	31040	2.97	11	4.81	x		x
**Tryptophan**	(K)ESAFEFLSSAWLDNDVTK(C)	20765	3.00	7	11.07	x	x	x
**Cysteine**	(K)ESAFEFLSSACLDNDVTK(C)	1455	-1.32	4	15.62	x		x
**Glutamine**	(K)ESAFEFLSSAQLDNDVTK(C)	836	-6.67	2	16.79			
**Phenylalanine**	(K)ESAFEFLSSAPLDNDVTK(C)	816	-2.60	3	5.03			

Supplementary Table S1. Mass spectrometry detection of amino acids encoded by stop codon. Intensity (in arbitrary units, a. u.), relative precursor mass deviation (ΔM), fragment count (b-y ions) and fragment mass deviation (RMS error) of peptides detected in our study and table of amino acids identified in other studies that used eukaryotic translation systems.

**PST number**	**Name**	**Source**
	pEYFP-C1	Thermo Scientific
	pcDNA3.1/Myc-His(−)A	Thermo Scientific
	pEXP-N-Venus	Anja Muntau lab
	pENTR/D-TOPO	Thermo Scientific
1226	pEXP-N-Venus-PTS1-ACOX3	(Schueren et al., 2014)
1360	pDRVL	(Schueren et al., 2014)
1385	pDRVL-LDHB-SCC	(Schueren et al., 2014)
1435	pDRVL-MDH1-SCC	(Schueren et al., 2014)
1436	pEYFP-C1-MDH1x	This study
1443	pcDNA3.1-HA-MDH1x-myc	This study
1473	pDRVL-MDH1 [TAA CTA]	This study
1474	pDRVL-MDH1 [TAG CTA]	This study
1475	pDRVL-MDH1 [TAA TTA]	This study
1476	pDRVL-MDH1 [TGA TTA]	This study
1479	pDRVL-MDH1 [TGA CCA]	This study

1480	pDRVL-MDH1 [TGA CTT]	This study
1481	pDRVL-MDH1 [UGA CTG]	This study
1482	pEXP-N-Venus-PTS1-human	This study
1483	pEXP-N-Venus-PTS1-zebrafinch	This study
1502	pDRVL-MDH1 [UGA AUA]	This study
1508	pcDNA3.1-HA-MDH1x_TGG-myc	This study
1514	pEYFP-C1-MDH1TGGx	This study
1521	pDRVL-MDH1x	This study
1523	pDRVL-MDH1x_TGG	This study
1525	pDRVL-MDH1_Δ26	This study
1526	pDRVL-MDH1_Δ26_TGG	This study
1535	pEYFP-C1-MDH1TAAx	This study
1536	pEYFP-C1-MDH1TGGx_ΔL	This study
1581	pDRVL-MDH1x_scr	This study
1582	pDRVL-MDH1TGGx_scr	This study

Supplementary Table S2. DNA constructs used in this study.

**Oligo**	**Name**	**Sequence 5'-3'**
JH77	DR-MDH1-[TGAATA]_for	GTCACCGTTCCTCTGCCTGAATAGACAATGT
JH78	DR-MDH1-[TGAATA]_rev	CCGGACATTGTCTATTCAGGCAGAGGAACG
JH102	MDH1TGG_ΔL_rev	GCGCGGATCCTCAACGACATTTAGATTCTTCAGC
JH103	MDH1TAA_for	GTGCTTTTGAATTTCTTTCCTCTGCCTAACTAGACAATGATGTTACTAAATGCTTC
JH104	MDH1TAA_rev	GAAGCATTTAGTAACATCATTGTCTAGTTAGGCAGAGGAAAGAAATTCAAAAGCAC
JH105	DR-MDH1x_for	GTCACCGTTCCTCTGCCTGACTAGACAATGATGTTACTAAATGCTTCAAAGCTGAAGAATCTAAATGTCGTCTTGT
JH106	DR-MDH1x_rev	CCGGACAAGACGACATTTAGATTCTTCAGCTTTGAAGCATTTAGTAACATCATTGTCTAGTCAGGCAGAGGAACG
JH109	DR-MDH1TGGx_for	GTCACCGTTCCTCTGCCTGGCTAGACAATGATGTTACTAAATGCTTCAAAGCTGAAGAATCTAAATGTCGTCTTGT
JH110	DR-MDH1TGGx_rev	CCGGACAAGACGACATTTAGATTCTTCAGCTTTGAAGCATTTAGTAACATCATTGTCTAGCCAGGCAGAGGAACG
JH113	DR-MDH1xΔ26_for	GTCACCGTTCCTCTGCCTGACTAGACAATGATGTTACTAAATGCTTCAT

JH114	DR-MDH1xΔ26_rev	CCGGATGAAGCATTTAGTAACATCATTGTCTAGTCAGGCAGAGGAACG
JH137	DR-MDH1x-scr_for	GTCACCGTTCCTCTGCCTGACTAGACTTCAGTGACAACATAAGTACTTA TTATAGTCTAGTAATTATCAGGGCAGT
JH138	DR-MDH1x-scr_rev	CCGGACTGCCCTGATAATTACTAGACTATAATAAGTACTTAT GTTGTCACTGAAGTCTAGTCAGGCAGAGGAACG
JH139	DR-MDH1TGGx-scr_for	GTCACCGTTCCTCTGCCTGGCTAGACTTCAGTGACAACATAAGTACTTA TTATAGTCTAGTAATTATCAGGGCAGT
JH140	DR-MDH1TGGx-scr_rev	CCGGACTGCCCTGATAATTACTAGACTATAATAAGTACT TATGTTGTCACTGAAGTCTAGCCAGGCAGAGGAACG
OST1192	EcoRI-MDH1x_for	GCGCGAATTCTATGTCTGAACCAATCAGAG
OST1193	MDH1x-BamHI_rev	GCGCGGATCCTCAAAGACGACATTTAGATTCT
OST1204	NheI-HA tag-MDH1	GCGCGCTAGCATGTACCCATACGATGTTCCAGATTACGCTTCTGAACCA ATCAGAGTCC
OST1205	BamHI-MDH1x-wo_second_STOP	GCGCGGATCCAAGACGACATTTAGATTCTTC
OST1231	MDH1TGG-mut_for	GCTTTTGAATTTCTTTCCTCTGCCTGGCTAGACAATGATGTTAC
OST1232	MDH1TGG-mut_rev	GTAACATCATTGTCTAGCCAGGCAGAGGAAAGAAATTCAAAAGC
OST1245	MDH1-human-PTS1_for	CACCTTCAAAGCTGAAGAATCTAAATGTCGTCTTTGA
OST1246	MDH1-human-PTS1_rev	TCAAAGACGACATTTAGATTCTTCAGCTTTGAAGGTG
OST1247	MDH1-zebra finch-PTS1_for	CACCTTCAGAGTGGAAGAATCGAAAAGCCGTCTGTAG
OST1248	MDH1-zebra finch-PTS1_rev	CTACAGACGGCTTTTCGATTCTTCCACTCTGAAGGTG
OST1463	DR-MDH1-[TAA CTA]_for	GTCACCGTTCCTCTGCCTAACTAGACAATGT
OST1464	DR-MDH1–[TAA CTA]_rev	CCGGACATTGTCTAGTTAGGCAGAGGAACG
OST1465	DR-MDH1–[TAG CTA]_for	GTCACCGTTCCTCTGCCTAGCTAGACAATGT
OST1466	DR-MDH1–[TAG CTA]_rev	CCGGACATTGTCTAGCTAGGCAGAGGAACG
OST1467	DR-MDH1–[TAA TTA]_for	GTCACCGTTCCTCTGCCTAATTAGACAATGT
OST1468	DR-MDH1–[TAA TTA]_rev	CCGGACATTGTCTAATTAGGCAGAGGAACG
OST1469	DR-MDH1–[TGA TTA]_for	GTCACCGTTCCTCTGCCTGATTAGACAATGT
OST1470	DR-MDH1–[TGA TTA]_rev	CCGGACATTGTCTAATCAGGCAGAGGAACG
OST1475	DR-MDH1–[TGA CCA]_for	GTCACCGTTCCTCTGCCTGACCAGACAATGT
OST1476	DR-MDH1–[TGA CCA]_rev	CCGGACATTGTCTGGTCAGGCAGAGGAACG
OST1477	DR-MDH1–[TGA CTT]_for	GTCACCGTTCCTCTGCCTGACTTGACAATGT
OST1478	DR-MDH1–[TGA CTT]_rev	CCGGACATTGTCAAGTCAGGCAGAGGAACG
OST1479	DR-MDH1–[TGA CTG]_for	GTCACCGTTCCTCTGCCTGACTGGACAATGT
OST1480	DR-MDH1–[TGA CTG]_rev	CCGGACATTGTCCAGTCAGGCAGAGGAACG

Supplementary Table S3. Oligonucleotides used in this study. DR, dual reporter.
